# Prediction of Pathological Complete Response to Neoadjuvant Chemotherapy for Primary Breast Cancer Comparing Interim Shear Wave Elastography Versus Magnetic Resonance Imaging

**DOI:** 10.1002/jum.16765

**Published:** 2025-07-08

**Authors:** Alessandro Garlaschi, Nicole Brunetti, Simona Tosto, Licia Gristina, Piero Fregatti, Massimo Calabrese, Alberto Stefano Tagliafico

**Affiliations:** ^1^ Department of Radiology IRCCS – Ospedale Policlinico San Martino Genoa Italy; ^2^ Department of Experimental Medicine (DIMES) University of Genova Genoa Italy; ^3^ Department of Surgical Sciences and Integrated Diagnostic (DISC) School of Medicine, University of Genova Genoa Italy; ^4^ Breast Surgery Clinic IRCCS Ospedale Policlinico San Martino Genoa Italy; ^5^ Department of Health Sciences (DISSAL) University of Genova Genoa Italy

**Keywords:** breast cancer, magnetic resonance imaging, neoadjuvant chemotherapy, pathological complete response, shear wave elastography

## Abstract

**Objectives:**

The prediction of pathological complete response (pCR) of primary breast cancer to neoadjuvant chemotherapy (NAC) is crucial for guiding surgical decisions.

This study examined the effectiveness of shear wave elastography (SWE) compared with magnetic resonance imaging (MRI) in predicting the response to NAC in patients with breast lesions.

**Methods:**

This prospective, single‐center observational study enrolled 110 patients diagnosed with BC who received NAC from January 2022 to May 2024. All patients underwent breast MRI, US, and SWE both at baseline and after treatment.

The treatment response was evaluated using the Miller–Payne (MP) classification, which assigns a score from G1 (no response) to G5 (complete response, absence of malignant cells).

**Results:**

The results showed that 43.64% (48/110) of patients achieved a pathological complete response (pCR), while 56.36% (62/110) had a partial response (NpCR). Tissue stiffness analysis using SWE revealed a significant reduction in stiffness, with an average decrease of 10 kPa in more than 50% of patients. Thresholds of 10, 20, 30, and 50 kPa were evaluated to assess their predictive value for pCR. All thresholds demonstrated statistically significant discriminative power (*P* < .0001), with AUCs of 0.675 (Se 51.6%, Sp 83.3%), 0.751 (Se 70.97%, Sp 79.17%), 0.749 (Se 79.03%, Sp 70.83%) and 0.677 (Se 85.5%, Sp 50.0%), respectively.

**Conclusions:**

The Shear Wave Elastography  system, which measures tissue stiffness through the speed of shear wave propagation, has proven to be a promising method for monitoring the response to chemotherapy, providing quantitative information that can complement other diagnostic methods such as magnetic resonance.

**Abbreviations**
ABC: advanced breast cancer
BC: breast cancer
LABC: locally advanced breast cancer
MRI: magnetic resonance
NAC: neoadjuvant chemotherapy
pCR: pathological complete response
ROI: region‐of‐interest
SWE: shear wave elastography

Breast cancer (BC) is the most prevalent malignancy worldwide and represents the second leading cause of death among women.^1^ Advanced breast cancer (ABC), including locally advanced breast cancer (LABC) and metastatic breast cancer, is characterized by a high incidence of metastasis and poor prognosis, often precluding initial surgical intervention. Consequently, the primary objectives of treatment for ABC are to delay disease progression, extend survival, and enhance patients' quality of life.^2^, ^3^

Neoadjuvant chemotherapy (NAC) plays a crucial role in the management of breast cancer by downstaging tumors, enabling less extensive breast and axillary surgery, and allowing monitoring of treatment response. The benefits of NAC include a reduction in tumor burden, an increased likelihood of breast and axillary conservation surgery, and improved survival outcomes, particularly for patients achieving a pathological complete response (pCR) in the breast and/or axilla. However, a subset of patients presents residual disease post‐NAC, which has significant implications for subsequent adjuvant therapy decisions.^4^

The assessment of NAC response is conventionally guided by RECIST criteria, using imaging modalities such as ultrasound (US) and magnetic resonance imaging (MRI). Among these, dynamic contrast‐enhanced MRI is regarded as the most accurate technique for evaluating tumor response post‐NAC.^5^ pCR, defined as the absence of microscopic evidence of invasive tumor cells following NAC, is associated with improved disease‐free and overall survival. Early prediction of pCR can influence surgical planning for both the breast and axilla, underscoring the clinical significance of accurate response assessment.^6^, ^7^, ^8^

Tumor histotype and biology are critical factors affecting MRI accuracy after NAC. Less aggressive subtypes, such as hormone receptor‐positive or lobular carcinomas, typically exhibit lower angiogenic activity and reduced contrast enhancement, which may complicate the interpretation of imaging findings in cases of pCR.^5^, ^9^ Despite advancements in diagnostic imaging, the pathological evaluation of surgical specimens remains the gold standard for detecting residual disease. However, even with negative imaging results, a residual disease is often identified with the examination of surgical specimens, highlighting the need for more reliable diagnostic imaging techniques. Moreover, MRI has several disadvantages, including being time‐consuming and costly, requiring the administration of an intravenous contrast agent, and being unsuitable or contraindicated for certain patients.^10^, ^11^

Shear‐wave elastography (SWE) is an ultrasound imaging technique that allows for reproducible and non‐invasive quantification of tissue elasticity (stiffness) and is well tolerated by all patients, even in pathological contexts such as fibrosis and cancer. Unlike conventional elastography, SWE employs focused radiation forces without manual compression, reducing operator dependency and improving reproducibility.^4^ Recent studies have described both qualitative and quantitative SWE methodologies, utilizing color maps and measurements of either pressure (kPa) or elastic wave speed (m/s) in analyses.^1^, ^12^

Given the relationship between tumor stiffness and stromal collagen content, SWE offers potential utility in evaluating structural tumor abnormalities before and after NAC. We hypothesize that SWE can provide valuable insights into tumor response to NAC by measuring changes in stromal stiffness, thereby complementing current imaging techniques.^5^

The aim of our study was to assess the accuracy of SWE in detecting residual breast cancer post‐NAC, comparing its diagnostic performance with that of MRI, currently considered the gold standard in clinical practice. Postoperative pathological outcomes served as the reference standard for evaluating the reliability of these diagnostic modalities.^11^

## Materials and Methods

### Patients

The study was approved by the local Ethics Committee of our Hospital (009REG2018).

This is a prospective, single‐center observational study. Written informed consent was obtained from all patients.

We consecutively enrolled patients with a diagnosis of BC diagnosed by ultrasound‐guided core needle biopsy who received NAC from January 2022 to May 2024.

All patients underwent breast MRI before starting and after the end of NAC.

All included patients also underwent SWE following both breast MRI and subsequently underwent surgical intervention at our center to evaluate pathological response, which served as the reference standard.

The following exclusion criteria were applied:

- Inability to obtain informed consent from the patient.
- Patients with respiratory difficulties.
- Beginning NAC more than 10 days before study enrollment.
- Patients who have not completed NAC.
- Lesion locations at suboptimal depths (either too superficial or too deep) potentially compromise the accuracy of SWE measurements.
- Presence of breast implants, which may affect the assessment of tissue elasticity.
- Lesions with marked edema or inflammation, potentially affecting stiffness measurements.
- Overlying skin abnormalities (eg, scars, ulcers) that could impede proper SWE data acquisition.

### Shear‐Wave Elastography Data Acquisition

Conventional B‐mode and shear wave elastography (SWE) acquisitions were performed using the Samsung RS85 ultrasound with a 3.0–12.0 MHz ultrasound probe. Conventional US and SWE were performed for each patient at baseline before NAC and at the end of NAC. The SWE probe was applied to the breast lesion and held steady for a few seconds to ensure the capture of high‐quality SWE images, which were then frozen and saved. The region of interest (ROI) box on the color map encompassed the entire lesion and surrounding normal tissue, as visualized on the B‐mode image. The tissue stiffness of each pixel within the image was displayed as a semi‐transparent color map overlaid on the gray‐scale image. Generally, the color scale ranged from 0 (dark blue, indicating the lowest stiffness) to red (indicating the highest stiffness, up to 180 kPa). Ten regions of interest (ROIs) were manually drawn on the color map to focus on the stiffest portion of the lesion. The elasticity parameters were automatically calculated. The mean stiffness in kilopascals (kPa) was taken as the average of the values taken from SWE images acquired on two orthogonal planes and recorded in our electronic database. SWE was performed by two radiologists with 6 and 15 years of experience in breast imaging (N.B. and A.G.), following both MRI examinations. Both radiologists were blinded during the SWE examination to the histopathologic, clinical, and MRI findings.

### Breast MRI

All MRI examinations were performed using a clinical 1.5 T MRI scanner (Siemens Magnetom Aera 1.5 Tesla, Siemens Healthcare, Erlangen, Germany). The standard MRI protocol included the following sequences: localizing sequence; fat‐saturated T2‐weighted imaging; diffusion‐weighted images acquired before contrast‐agent injection using a single‐shot echo‐planar imaging (SE‐EPI) sequence (TR/TE = 2496/71 ms, slice thickness = 5 mm, slice spacing = 1 mm, *b*‐value = 0/800 s/mm^2^); three‐dimensional fat‐suppressed gradient‐echo T1‐weighted sequences (T1‐flash3D, TR = 4.33 ms, TE = 173.52 ms; matrix = 768 × 768); 340 × 340 mm field of view with dynamic field of view; 0.9 mm isotropic resolution; acquisition time <10 min; echo train length: 1; number of excitations: 1, both before and after bolus injection of gadolinium‐based contrast medium (gadobenate dimeglumine, MultiHance, Bracco Imaging, Milan, Italy; 0.1 mmol/kg body weight; injection rate of 2.0 mL/s, followed by a 20 mL saline flush). On axial planes, subtraction images were obtained from the contrast‐enhanced and unenhanced images. Maximum‐intensity‐projection images were then reconstructed using the first subtracted contrast‐enhanced dynamic sequence. Early post‐contrast T1‐weighted sequences were used to obtain measurements of tumor at baseline and after NAC, and RECIST criteria were used to assign the patient to assessment categories.

### Pathological Assessment

Pathological response to NAC was assessed using the Miller–Payne (MP) classification on surgical specimens. The MP system categorizes response into five grades:

- G1: No change or minimal alterations in individual malignant cells, with no overall reduction in cellularity.
- G2: Minor loss of tumor cells, with up to 30% reduction in overall cellularity.
- G3: Estimated tumor cell reduction between 30 and 90%.
- G4: Marked tumor cell loss, with only small clusters or scattered individual cells remaining (~90% reduction).
- G5: No identifiable malignant cells in the tumor site; only fibroelastic vascular stroma remains, often containing macrophages. Ductal carcinoma in situ may still be present.

Patients were categorized into two groups based on their MP classification: non‐pCR (NpCR) group (G1–G4) and pCR group (G5).

## Statistical Analyses

MedCalc version 12.7.0 (MedCalc Software, Mariakerke, Belgium) was employed for the statistical analyses. Means with standard deviation were used for continuous variables and SWE parameters; categorical variables are reported as percentages and numbers. Stiffness and delta (Δ) stiffness between pCR and NpCR with different cut‐offs were compared by using Student's t test. Areas under the ROC curves (AUC) were compared utilizing the DeLong method to estimate the diagnostic performance of SWE and compared with MRI to predict NAC. We analyzed the following diagnostic performance parameters—sensitivity, specificity, positive likelihood ratio (+LR), and negative likelihood ratio (−LR) for all evaluated cut‐off values (10, 20, 30, and 50 kPa).

## Results

The mean age of the 110 patients was 53.61 years (range: 25–86 years).

The pre‐NAC MRI evaluation showed lesions smaller than 3 cm in 55.45% of cases (61 patients) and larger than 3 cm in 44.55% of cases (49 patients).

The definitive assessment of the response to NAC was based on the histopathological analysis of post‐operative samples, using the MP classification criteria.

The pCR rate was 43.64% (48 patients), and the NpCR rate was 56.36% (62 patients).

The post‐NAC MRI evaluation showed a complete response in 50% of cases (55 patients), while 50% of cases (55 patients) showed residual disease (Table 1).

**Table 1:** Clinical and Histological Features of Patients Included in Our Investigation

Clinical Data and Pathologic Result for Patients With Breast Cancer	
Age, years	
Median	53.61
Range	25–86
Tumor size (cm)	3.56 (0.9–12)
Tumor size, n (%)	
*x* ≤ 3 cm	61 (55.45%)
*x* > 3 cm	49 (44.55%)
HER2 status, n (%)	
Positive	45 (40.91%)
Negative	65 (59.09%)
Ki‐67 index, n (%)	
≥15%	82 (74.55%)
<15%	28 (25.45%)
Grading, n (%)	
G3	14 (12.73%)
G2	44 (40%)
G1	4 (3.64%)
G0	48 (43.64%)

**Figure 1:**
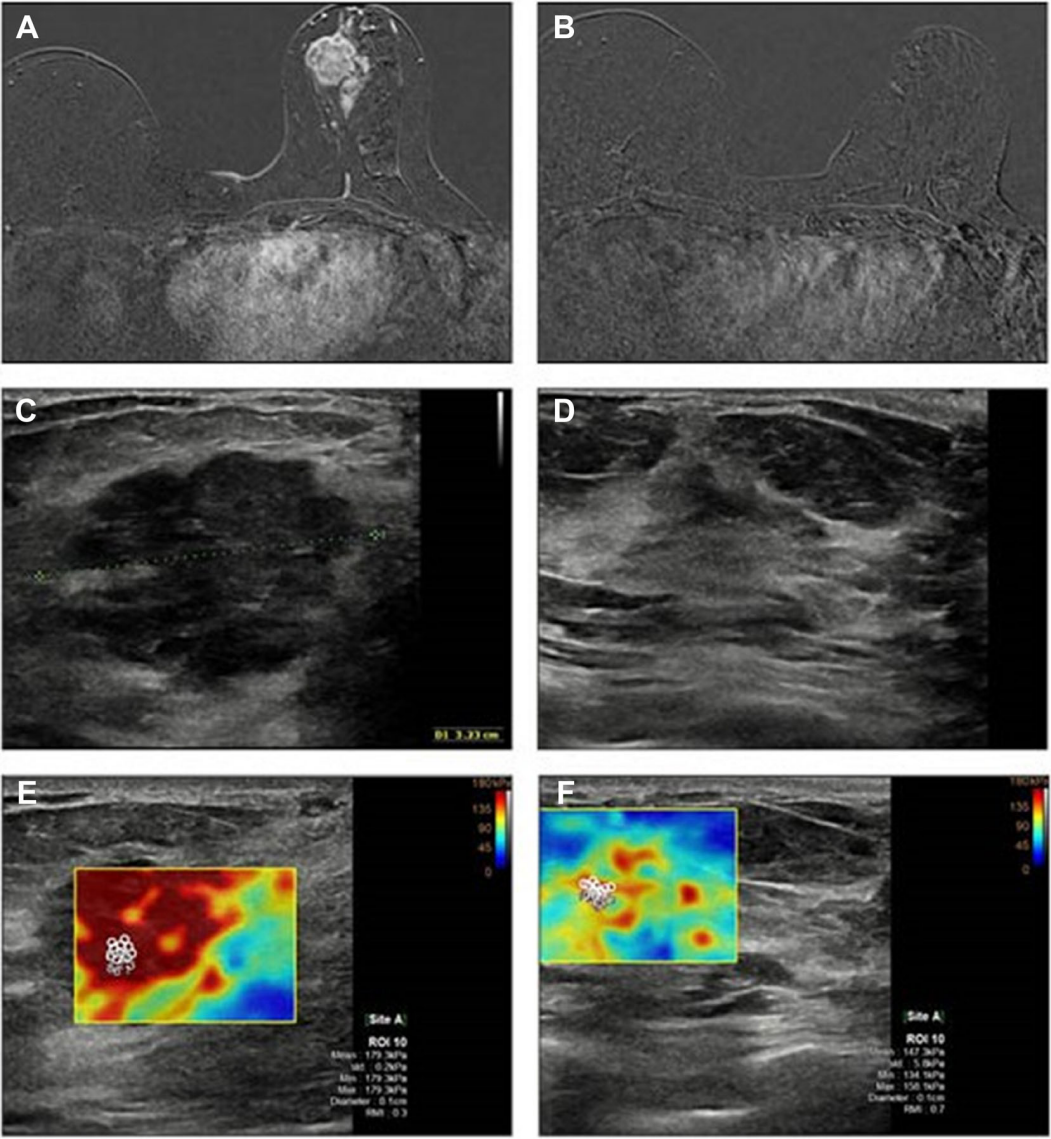
Images showing baseline pre‐NAC and post‐NAC MRI (**A**, **B**), US (**C**, **D**), and SWE (**E**, **F**) in a woman who had residual invasive cancer correctly predicted by SWE and US, but not by MRI. T1‐weighted, contrast‐enhanced MRI image showing appearance of complete response post‐NAC, in the absence of areas characterized by contrast‐enhancement (**B**). On the contrary, US and SWE images demonstrate a persistence of pathology with a reduction in size (**D**) and a reduction in tissue stiffness (**F**) compared with the pre‐NAC investigation. The definitive post‐surgery histological examination demonstrated residual disease.

**Figure 2:**
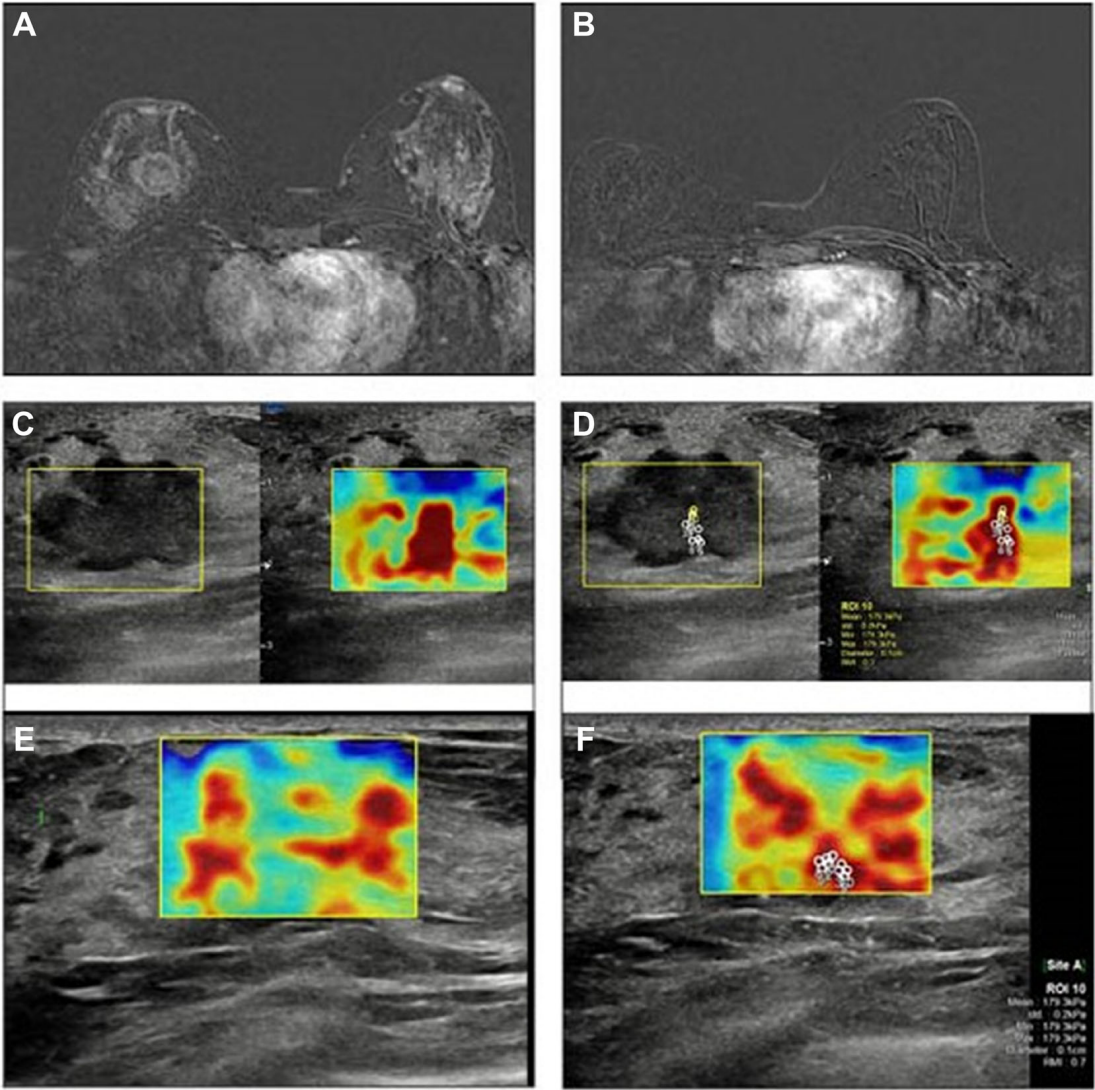
Images showing baseline pre‐NAC and post‐NAC MRI (**A**, **B**), US (**C**, **D**), and SWE (**E**, **F**) who had residual invasive cancer did not predict correctly by MRI. In the T1‐weighted sequences, there are no areas of contrast‐enhancement in the post‐contrast sequences. In the SWE images, there is a marked reduction in tissue stiffness (>10%) with a difference of ~30 kPa. An incomplete pathological response was demonstrated at post‐surgery histological examination.

The post‐NAC SW evaluation showed a 10% reduction in tissue stiffness, measured in kPa, in 52.73% of cases (58 patients) and a 5% reduction in 68.18% of cases (75 patients), with an absolute decrease in kPa values in 89.09% of cases (98 patients) (Figures 1 and 2).

The SW evaluation demonstrated changes in tissue elasticity corresponding to the tumor, allowing a comparison between values before the start of NAC and at the end of the treatment cycle.

Several DELTA values were considered:

- DELTA CUT‐OFF 10 kPa.
- DELTA CUT‐OFF 20 kPa.
- DELTA CUT‐OFF 30 kPa.
- DELTA CUT‐OFF 50 kPa.

These were used to compare the MRI results and the final histological evaluation.

Statistical analysis demonstrated that both MRI and SW evaluations significantly predicted the response to NAC.

A difference of 10 kPa in SWE evaluation before and after NAC was the threshold most closely associated with the histopathological outcome, particularly due to its highest specificity in detecting residual disease. All thresholds demonstrated strong predictive power, with statistically significant results.

Similarly, the other CUT‐OFF values considered in the statistical analysis correlated with pCR.

ROC analysis showed the following AUC measurements for predicting pathological complete response at the end of NAC:

- MRI tumor size reduction: AUC = 0.759
- SWE Δ ≥10 kPa: AUC = 0.675 (95% CI: 0.579–0.761), Sensitivity = 51.61%, Specificity = 83.33%
- SWE Δ ≥20 kPa: AUC = 0.751 (95% CI: 0.659–0.828), Sensitivity = 70.97%, Specificity = 79.17%
- SWE Δ ≥30 kPa: AUC = 0.749 (95% CI: 0.658–0.827), Sensitivity = 79.03%, Specificity = 70.83%
- SWE Δ ≥50 kPa: AUC = 0.67 (95% CI: 0.582–0.763), Sensitivity = 85.5%, Specificity =50.0%.

**Figure 3:**
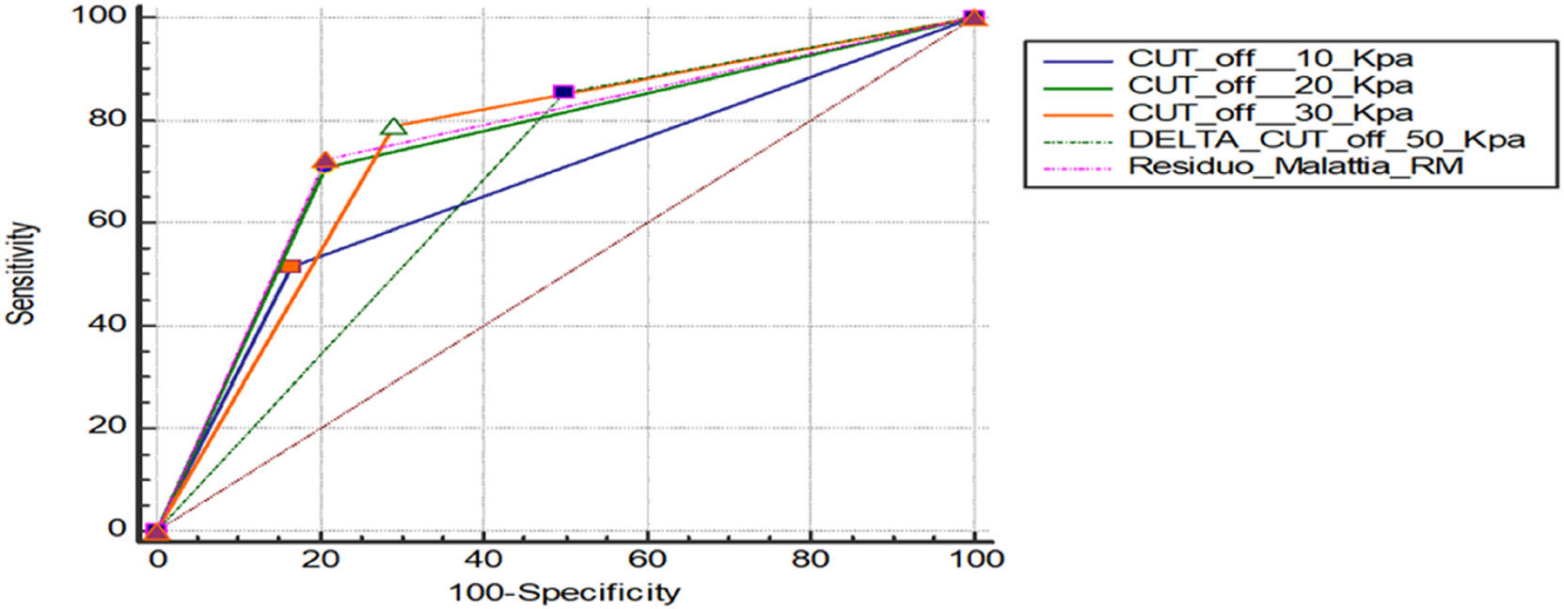
ROC curve demonstrating the relationship between the different delta cut‐off in kPa and MRI pathological residue.

All of the ROC curves shown on one graph are shown in Figure 3.

## Discussion

**Figure 4:**
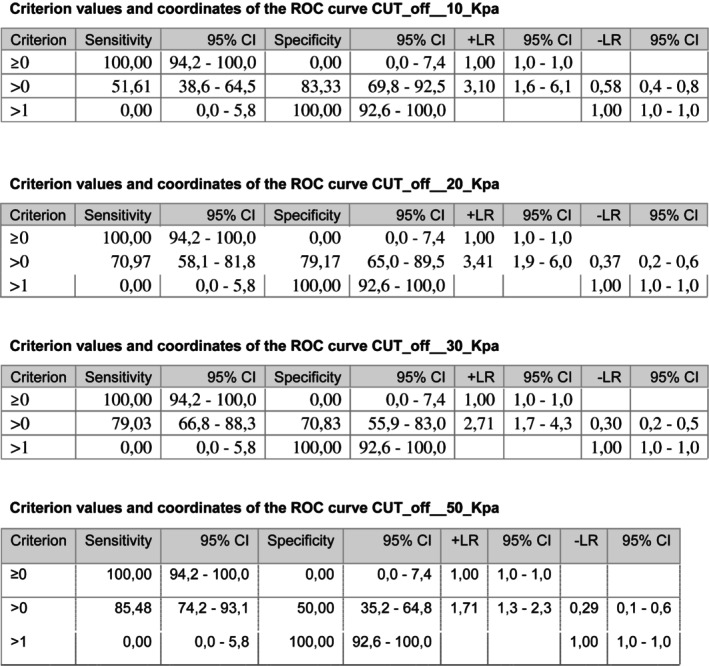
Criterion values and coordinates of the ROC curves for SWE stiffness reductions of 10, 20, 30, and 50 kPa.

SWE, a new technology in clinical applications in recent years, can provide quantitative information by measuring the stiffness of breast masses, providing quantitative information of tissue stiffness (elasticity) from the speed of shear wave propagation in tissues. The decrease in tumor stiffness during treatment is related to the curative effect. Shear waves (transverse waves) are generated by the acoustic radiation force of a focused ultrasound (US) beam passing into the breast. They travel faster in stiff tissue compared with soft tissue. By capturing the propagation of the shear waves, an elasticity map is produced. This is a color overlay of the US image, with different colors representing the speed of the shear waves (in m/s) or the tissue stiffness (Young modulus [*E*] in kilopascals or kPa). From the elasticity map, the stiffness of a mass can be assessed from the color score or the color pattern, or from quantitative parameters. SWE allows for quantitative assessment of stiffness of a mass, and as such, objective correlation between prognostic features of breast cancer and quantitative values of its stiffness can be performed. Compared with those in soft tissue, the waves in hard tissue travel faster, which leads to higher vs values. A high degree of hardness in the breast lesion increases the possibility of malignancy (Figure 4).^3^

In this study, SWE performed after NAC using a simple threshold of 20 kPa for mean elasticity was found to be associated with the likelihood of achieving pCR after NAC, yielding results comparable to those of MRI. Simple threshold assessment resulted in a strong association with pCR, with an area under the curve of around 0.75 for 20 kPa, 30 kPa, and MRI. The three approaches using 20 kPa, 30 kPa, and MRI did not show statistically significant differences, suggesting that SWE could be comparable to MRI to assess pCR after NAC.

A previous study has assessed the value of evaluating response to NAC with SWE at the end of NAC. They found that a threshold of 30 kPa was useful in predicting response. They found this cut‐off value more predictive than other higher values. They did not attempt to look at the value of percentage reduction in stiffness compared with baseline values.^13^

This study was able to demonstrate an overall change with a reduction in stiffness of the breast tumor, particularly in the peritumoral tissue, after NAC. The goal was also to show that a simple measurement technique using specific ROIs in the region of interest could improve the ability to predict the response to NAC.

The potential of SWE in predicting NAC response has been reported in recent years.^14^ The cellular and molecular mechanisms by which the extracellular matrix affects tissue stiffness have been illustrated by previous basic research. Crosslinking of collagen, one of the most essential components of the extracellular matrix, was revealed to modulate tissue stiffness. Meanwhile, at the molecular level, lysyl oxidase, fibronectin, and caveolin are responsible for this process, as mentioned before. More details suggest that lysyl oxidase accelerates the motility and invasion of cancer cells, while lack of caveolin not only results in early tumor recurrence but also leads to metastasis and drug resistance.^15^

Consequently, one of the main issues related to SWE is the possibility of a residual mass of fibrous tissue post‐NAC, which can be measured as tumor residue; however, fibrous tissue has characteristics that are still softer than those of the tumor Additional limitations of SWE include the difficulty in assessing very deep lesions in the breast and in patients with respiratory difficulties.

Moreover, the exclusion of patients with suboptimally deep lesions or breast implants may have introduced a selection bias, potentially limiting the generalizability of the results.

It is becoming more common to see pCR in breast cancer as NAC regimens become more effective, resulting in more women undergoing surgical resection of breast tissue even though no viable tumor is present.^16^

It is also becoming clear that different immunophenotypes of breast cancer have different rates and patterns of response to NAC and that this necessitates a tailored approach by the radiologist when assessing response to NAC.^17^

The main limitations of our study are the modest number of patients and the data derives from a single hospital center; furthermore, no differentiation is made based on histotype or tumor prognostic characteristics.

The study suggests that SWE may offer a comparable level of accuracy to MRI based on RECIST criteria and has certain advantages over MRI, such as faster execution, lower cost, and should be considered in patients with contraindications to MRI (claustrophobia, metallic devices).

In conclusion, SWE appears to be a promising tool for predicting pathological response in breast cancer patients undergoing neoadjuvant chemotherapy. Although the initial results are encouraging, larger and multicenter studies are necessary to validate these findings.

## Data Availability

The data that support the findings of this study are available from the corresponding author upon reasonable request.
